# Oestrogen receptor low positive breast cancer: associations with prognosis

**DOI:** 10.1007/s10549-023-07040-9

**Published:** 2023-07-18

**Authors:** Anette H. Skjervold, Marit Valla, Anna M. Bofin

**Affiliations:** 1 Department of Clinical and Molecular Medicine, Faculty of Medicine and Health Sciences, Norwegian University of Science and Technology, Trondheim, Norway; 2 Department of Pathology, St. Olav’s Hospital, Trondheim, Norway

**Keywords:** Breast cancer, Oestrogen receptor, ER, ER low positive, Prognosis, Endocrine treatment

## Abstract

**Purpose:**

In this study of oestrogen receptor (ER) Low Positive breast cancers (BC) in three large cohorts of BC patients, we assess associations between levels of ER expression and tumour characteristics and prognosis.

**Methods:**

Cases were stratified into patients unlikely to have received adjuvant therapy according to treatment guidelines at time of diagnosis (before 1995), and those who could have received adjuvant therapy (diagnosed in 1995 or later). ER status was divided into < 1%; ≥ 1 < 10%; ≥ 10%. Results were correlated with time of diagnosis, histopathological grade, proliferation status, and molecular subtypes, using Pearson’s Chi-square test. For prognosis, hazard ratios and cumulative incidence of death from BC were used.

**Results:**

Of the 1955 tumours, 65 (3.3%) were ER Low Positive (ER ≥ 1 < 10%). Overall, the highest proportion of ER Low Positive tumours was observed among Luminal B (HER2 +) subtype (9.4%) and grade 3 tumours (4.3%). The risk of death from BC was lower in ER Low Positive and ER ≥ 10% compared to ER-negative cases. Compared to patients diagnosed before 1995, women diagnosed in 1995 or later showed a higher proportion of ER Low Positive BCs, and their tumours were of smaller size, lower grade, and lower proliferative status. There was no significant difference in prognosis compared to those with ER ≥ 10% tumours.

**Conclusion:**

Women with ER Low Positive tumours diagnosed in a time period when adjuvant therapy was available had tumours of smaller size, lower grade, and lower proliferative status, and similar prognosis to those with ER ≥ 10% compared to women diagnosed earlier.

## Introduction

Oestrogen receptor (ER) status plays an essential role in clinical decision-making and predicting outcome and treatment response for breast cancer (BC) patients [1]. According to current guidelines [2], patients with ER-positive tumours are considered eligible for endocrine therapy. Patients with ER-negative tumours are more likely to benefit from chemotherapy and generally have a poorer outcome than patients with ER-positive (ER +) tumours [3, 4].

Breast cancer differs from most tumours because of its dependence on female sex hormones for development and growth [5]. Expression of ER by immunohistochemistry (IHC) is seen in more than 70% of BC tumours [6]. The ASCO/CAP and current national BC guidelines state that BC tumours with ≥ 1% positive staining tumour cell nuclei should be interpreted as ER + , and negative if < 1% of tumour cell nuclei express ER [2, 7]. However, the ASCO/CAP Expert Panel states that data on the effect of endocrine therapy for cancers with ER ≥ 1 < 10% are limited. They suggest that samples with ER ≥ 1 < 10% should be reported as ER Low Positive, with a comment mentioning the limited data available on the therapeutic benefit of anti-hormonal treatment for this group of patients [2]. According to the St. Gallen 2019 Consensus Discussion on The Optimal Primary Breast Cancer Treatment, there is a need for better evaluation of ideal cut-off levels for the prescription of endocrine therapy for ER + tumours, particularly for ER Low Positive cases [8–10].

In this study we examined expression levels of ER in BC tumours and associations between ER status and time of diagnosis, and tumour characteristics such as histopathological grade, molecular subtypes, proliferation and prognosis, with emphasis on ER Low Positive tumours.

## Materials and methods

### Study population

**Fig. 1:**
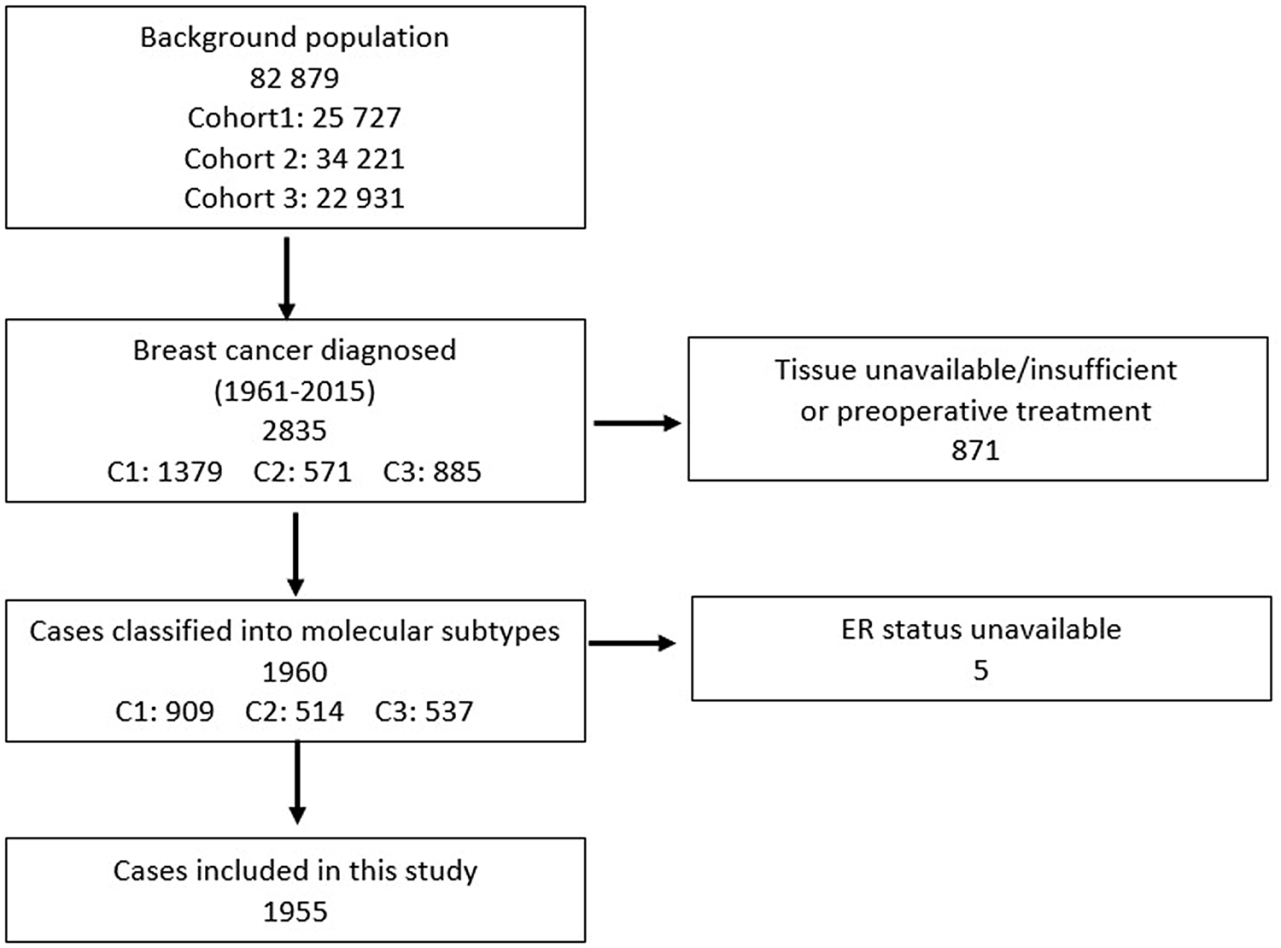
Overview of the three cohorts of breast cancer patients included in the study

This study comprises women from three population-based surveys conducted in Trøndelag County, Norway. Information on breast cancer incidence was obtained from the Cancer Registry of Norway. Date of death, and/or emigration was obtained from the National Population Register and causes of death from the Norwegian Cause of Death Registry. Formalin-fixed, paraffin embedded (FFPE) tumour tissue from the primary tumours and corresponding pathology reports were retrieved from the Department of Pathology at St. Olav’s Hospital, Trondheim University Hospital, Norway (Fig. 1).

Cohort 1: The cohort includes 25,727 women born 1886–1928 [11] invited to attend a population-based survey for the early detection of breast cancer conducted in Nord-Trøndelag County, Norway, between 1956 and 1959. During 47 years of follow-up (1961 to end of 2008), 1379 new BCs were registered among these women. In a previous study 909 of these tumours were classified according to histopathological type and grade and divided into molecular subtypes [12]. For one case ER status was missing, and this case was excluded from the present study, leaving 908 cases. After diagnosis, patients were followed until time of death from BC or death from other causes, or until December 31st, 2015.

Cohort 2: The second cohort comprises 34,221 women born between 1897 and 1977 and derives from the HUNT2 Study conducted between 1995 and 1997 in Nord-Trøndelag County, Norway [13]. From attendance until December 31st, 2009, 728 women were diagnosed with BC. Of these, 157 cases were already included in Cohort 1 and 57 were unavailable for subtyping. The remaining tumours (*n* = 514) from Cohort 2 were assigned histopathological type and grade and reclassified into molecular subtypes [14]. ER status was available for all 514 cases. After diagnosis, these patients were followed until time of death from BC or death from other causes, or until December 31^st^, 2015.

Cohort 3: The third cohort includes 22,931 women born at E.C. Dahl’s Foundation, Trondheim, Norway between 1920 and 1966. During 52 years of follow-up (1961 to the end of 2012), a total of 870 women were diagnosed with BC. Among them, 598 were diagnosed at St Olav’s Hospital, and histopathological typing, grading and molecular subtyping were successful for 537 of these cases [15]. ER status was available for 533 of these cases. After diagnosis, patients were followed until time of death from BC or death from other causes, or until December 31st, 2015.

### Specimen characteristics

Tissue Microarray (TMA) paraffin blocks were made from the archival tumour tissue using the TissueArrayer Minicore with TMA Designer2 software (Alphelys). Three 1 mm in diameter tissue cylinders from the periphery of the FFPE primary tumours were transferred to TMA recipient blocks. TMA Sections (4 µm) were cut and IHC-staining for ER was carried out within four weeks after sectioning. Between cutting and staining, sections were stored at − 20 °C. Staining intensity was not quantified in this study. Molecular subtypes for all cases in all three cohorts were determined using IHC and in situ hybridization in lieu of gene expression analyses, and have been published previously [12, 14, 15]. The IHC markers including ER are shown in Table 1.

**Table 1:** Algorithm for reclassification of breast cancers into molecular subtypes [12]

Molecular subtype	Classified by
Luminal A	ER + and/or PR + , HER2-, Ki-67 < 15%
Luminal B (HER2-)	ER + and/or PR + , HER2-, Ki-67 ≥ 15%
Luminal B (HER2 +)	ER + and/or PR + , HER2 +
HER2 type	ER-, PR-, HER2 +
Five-negative phenotype	ER-, PR-, HER2-, CK5-, EGFR-
Basal phenotype	ER-, PR-, HER2-, CK5 + and/or EGFR +

*ER* Oestrogen receptor, *PR* Progesterone receptor, *HER2* Human epidermal growth factor receptor 2, *CK5* Cytokeratin 5, *EGFR* Epidermal growth factor receptor 1

### Statistical analyses

For the present study, we divided ER expression into three categories (< 1%; ≥ 1 < 10%; ≥ 10%) and studied associations between ER expression and histopathological grade, molecular subtype, proliferation, and prognosis.

Pearson’s chi square test was used to compare patient and tumour characteristics across categories of ER. In analyses of prognosis, we distinguished between women diagnosed before 1995 and women diagnosed in 1995 or later. This cut-off was used to approximate the gradual implementation of adjuvant treatment in Norway [14, 16]. Cumulative incidence of death from BC was estimated, with death from other causes as competing events. Gray’s test was used to compare equality between cumulative incidence curves. Cox proportional hazard analyses were used to estimate hazard ratios (HR) of BC death with 95% confidence intervals (CI) within each diagnostic period, censoring at time of death from other causes. We adjusted for age, stage, histopathological grade, and for these variables combined. No clear violations of proportionality were found in log-minus-log plots. Statistical analyses were performed using Stata/MP version 17 (StataCorp LP, College Station, Texas, USA).

## Results

Patient and tumour characteristics for the 1955 patients included in the present study are shown in Table 2. Mean age at diagnosis was 67.3 years (SD: 12.8) and mean follow-up after diagnosis was 9.9 years (SD: 7.3). By end of follow-up, 545 (27.9%) patients had died from BC and 588 (30.1%) died from other causes. Of the 1955 tumours, 315 (16.1%) were ER < 1%, 65 (3.3%) were ER Low Positive (ER ≥ 1 < 10%) and 1575 (80.6%) were ER ≥ 10%. Of the 545 deaths from BC, 129 (23.7%) cases were ER < 1%, 16 (2.9%) were ER Low Positive and 400 (73.4%) were ER ≥ 10%.

**Table 2:** Patient and tumour characteristics according to ER categories

	Total study population	ER categories			
		< 1%	≥ 1 < 10%	≥ 10%	*p* value (χ^2^)
*N* (%)	1955	315 (16.1)	65 (3.3)	1575 (80.6)	
Mean age at diagnosis, years (SD)	67.3 (12.8)	65.4 (14.0)	63.3 (13.9)	67.9 (12.4)	
Mean follow-up, years (SD)	9.9 (7.3)	8.4 (7.6)	10.3 (6.9)	10.2 (9.0)	
Alive Dec. 31st 2015 (%)	822 (42.1)	102 (32.5)	34 (51.5)	686 (43.6)	< 0.001
Deaths from breast cancer (%)	545 (27.9)	129 (41.0)	16 (24.6)	400 (25.4)	
Deaths from other causes or by the end of 2015 (%)	588 (30.1)	84 (26.7)	15 (23.1)	489 (31.1)	
*Histopathological grade (%)*					
I	287 (14.7)	13 (4.1)	6 (9.2)	268 (17.0)	< 0.001
II	1015 (51.9)	73 (23.2)	31 (47.7)	911 (57.8)	
III	653 (33.4)	229 (72.7)	28 (43.1)	396 (25.1)	
*Tumour size (%)*					
≤ 2 cm	1035 (52.9)	124 (39.4)	33 (50.8)	878 (55.8)	< 0.001
> 2 cm, ≤ 5 cm	391 (20.0)	75 (23.8)	15 (23.1)	301 (19.1)	
> 5 cm	24 (1.2)	9 (2.9)	3 (4.6)	12 (0.8)	
Uncertain, but > 2 cm	161 (8.2)	44 (14.0)	7 (10.8)	110 (7.0)	
Uncertain	344 (17.6)	63 (20.0)	7 (10.8)	274 (17.4)	
*Stage (%)*					
I	881 (45.1)	113 (35.9)	25 (38.5)	743 (47.2)	0.010
II	708 (36.2)	137 (43.5)	26 (40.0)	545 (34.6)	
III	98 (5.0)	23 (7.3)	3 (4.6)	72 (4.6)	
IV	72 (3.7)	14 (4.4)	2 (3.1)	56 (3.6)	
Unknown	196 (10.0)	28 (8.9)	9 (13.9)	159 (10.1)	
*Molecular subtype (%)*					
Luminal A	937 (47.9)	10 (3.2)	29 (44.6)	898 (57.0)	< 0.001
Luminal B (HER2-)	552 (28.2)	12 (3.8)	19 (29.2)	521 (33.1)	
Luminal B (HER2 +)	180 (9.2)	7 (2.2)	17 (26.2)	156 (9.9)	
HER2 type	108 (5.5)	108 (34.3)	0 (0.0)	0 (0.0)	
5NP	53 (2.7)	53 (16.8)	0 (0.0)	0 (0.0)	
BP	125 (6.4)	125 (39.7)	0 (0.0)	0 (0.0)	
*Histopathological subtype (%)*					
Invasive carcinoma (NOS^a^)	1507 (77.1)	218 (69.2)	50 (76.9)	1239 (78.7)	< 0.001
Lobular carcinoma	210 (10.7)	17 (5.4)	8 (12.3)	185 (11.8)	
Tubular carcinoma	6 (0.3)	0 (0.0)	0 (0.0)	6 (0.4)	
Mucinous carcinoma	65 (3.3)	2 (0.6)	0 (0.0)	63 (4.0)	
Medullary carcinoma	60 (3.1)	38 (12.1)	4 (6.2)	18 (1.1)	
Papillary carcinoma	39 (2.0)	5 (1.6)	0 (0.0)	34 (2.2)	
Metaplastic	18 (0.9)	15 (4.8)	1 (1.5)	2 (0.1)	
Other	50 (2.6)	20 (6.4)	2 (3.1)	28 (1.8)	
*Ki-67 low/high (%)*					
Ki-67 < 15%	1057 (54.1)	74 (23.5)	31 (47.7)	952 (60.4)	< 0.001
Ki-67 ≥ 15%	898 (45.9)	241 (76.5)	34 (52.3)	623 (39.6)	
Mitoses/10 HPF, median (IQR p25, p75)	5 (2,13)	15 (7,29)	8 (4,17)	4 (1,10)	
*Mitoses/10 HPF, quartiles (%)*					
≤ 2	459 (23.5)	23 (7.3)	9 (13.9)	427 (27.2)	< 0.001
> 2, ≤ 5	275 (14.1)	23 (7.3)	6 (9.2)	246 (15.7)	
> 5, ≤ 13	342 (17.5)	54 (17.1)	10 (15.4)	278 (17.7)	
> 13	875 (44.9)	215 (68.3)	40 (61.5)	620 (39.5)	

^a^*NOS* Not otherwise specified

### ER categories and molecular subtypes

Of the 1955 tumours included in this study, 1669 (85.4%) were classified as one of the luminal subtypes (Luminal A, Luminal B (HER2-), or Luminal B (HER2 +)). Of these, 1640 were ER positive (ER ≥ 1%). Of the 180 cases of Luminal B (HER2 +), seven (3.9%) cases were ER < 1%, 17 (9.4%) were ER Low Positive and 156 (86.7%) were ER ≥ 10%  (*p* < 0.0001). Among the 937 cases with Luminal A subtype, 10 (1.1%) cases were ER < 1%, 29 (3.1%) were ER Low Positive and 898 (95.8%) were ER ≥ 10%. Of the 552 Luminal B (HER2-) cases 12 (2.2%) cases were ER < 1%, 19 (3.4%) were ER Low Positive and 521 (94.4%) were ER ≥ 10%. Twenty-six cases with ER < 1% were classified as Luminal based on progesterone receptor (PR) positivity (Table 2).

### ER categories, histopathological grade, proliferation, and histopathological type

In this study, 287 (14.7%) tumours were grade 1, 1015 (51.9%) were grade 2 and 653 (33.4%) were grade 3. The highest proportion of ER Low Positive (28/653 (4.3%)) was observed among grade 3 tumours (*p* < 0.0001). Of the 1057 cases with Ki-67 < 15%, 74 (7.0%) were ER < 1%, 31 (2.9%) were ER Low Positive, and 952 (90.1%) were ER ≥ 10%. Of the 898 cases with Ki-67 ≥ 15%, 241 (26.8%) were ER < 1%, 34 (3.8%) were ER Low Positive, and 623 (69.4%) were ER ≥ 10%  (*p* < 0.0001). Similarly, of the 459 cases with ≤ 2 mitoses/10 High power fields (HPF) (p25), 23 (5.0%) were ER < 1%, 9 (2.0%) were ER Low Positive and 427 (93.0%) were ER ≥ 10%  (*p* < 0.0001). Whereas, of the 875 cases with > 13 mitoses/10 HPF (p75), 215 (24.6%) were ER < 1%, 40 (4.5%) were ER Low Positive, and 620 (70.9%) were ER ≥ 10%  (*p* < 0.0001). Of the 65 ER Low Positive cases, 50/1507 (3.3%) were invasive carcinoma NOS, 8/210 (3.8%) were lobular carcinoma, 4/60 (6.6%) were medullary carcinoma, and 1/18 (5.5%) was metaplastic carcinoma (Table2).

### Comparisons between women diagnosed before 1995 and women diagnosed in 1995 or later

A total of 774 cases were diagnosed before 1995, and 1181 were diagnosed in 1995 or later. The distribution of cases according to time of diagnosis are shown in Table 3. Of women diagnosed before 1995, 352/774 (45.5%) died from BC during follow-up, as opposed to 193/1181 (16.3%) of those diagnosed in 1995 or later. Among women diagnosed before 1995, 152/774 (19.6%) tumours were ER < 1%, falling to 163/1181 (13.8%) among women diagnosed in 1995 or later. Similarly, 16/774 (2.1%) tumours were ER Low Positive before 1995, rising to 49/1181 (4.2%) in 1995 or later, and 606/774 (78.3%) cases diagnosed before 1995 were ER ≥ 10%, rising to 969/1181 (82.1%) among women diagnosed in 1995 or later. Furthermore, we found that 310/774 (40.1%) of tumours diagnosed before 1995 were ≤ 2 cm in diameter, rising to 725/1181 (61.4%) for tumours diagnosed in 1995 or later  (*p* < 0.0001) (Table 3).

**Table 3:** Patient and tumour characteristics among women diagnosed before 1995, or in 1995 and later

	Women diagnosed with BC before 1995 (%)	*p*-value	Women diagnosed with BC in 1995 or later (%)	*p*-value
Total cases (*n*)	774		1181	
Cohort 1 (*n* = 908)	661 (72.7)		248 (27.3)	
Cohort 2 (*n* = 514)	0 (0.0)		514 (100.0)	
Cohort 3 (*n* = 533)	113 (21.2)		420 (78.8)	
Mean age at diagnosis (SD)	69.5 (10.4)		65.4 (14.3)	
Mean follow-up-time (SD)	10.9 (9.7)		9.2 (5.0)	
Deaths by BC (%)	352 (45.5)	0.104	193 (16.3)	0.001
Deaths from other causes or by the end of 2015 (%)	364 (47.0)		224 (19.0)	
Alive at end of follow-up (31st Dec 2015)	58 (7.5)		764 (64.7)	
*Oestrogen receptor (%)*				
< 1% (%)	152 (19.6)	< 0.001	163 (13.8)	< 0.001
≥ 1 < 10% (%)	16 (2.1)		49 (4.2)	
≥ 10% (%)	606 (78.3)		969 (82.1)	
*Tumour size*				
≤ 2 cm (%)	310 (40.1)	0.023	725 (61.4)	< 0.001
> 2 ≤ 5 cm (%)	64 (8.3)		327 (27.7)	
Tumour size > 5 cm (%)	3 (0.4)		21 (1.8)	
Uncertain, but > 2 cm (%)	148 (19.1)		13 (1.1)	
Uncertain (%)	249 (32.2)		95 (8.0)	
*Stage*				
1	346 (44.7)	0.002	535 (45.3)	0.001
2	257 (33.2)		451 (38.2)	
3	47 (6.1)		51 (4.3)	
4	39 (5.0)		33 (2.8)	
Unknown	85 (11.0)		111 (9.4)	
*Histopathological grade*				
1	84 (10.9)	< 0.001	203 (17.2)	< 0.001
2	387 (50.0)		628 (53.2)	
3	303 (39.1)		350 (29.6)	
*Histopathological type*				
Invasive carcinoma (NOS)	566 (73.1)	< 0.001	941 (79.7)	< 0.001
Lobular carcinoma	96 (12.4)		114 (9.7)	
Mucinous carcinoma	27 (3.5)		38 (3.2)	
Medullary carcinoma	27 (3.5)		33 (2.8)	
Papillary carcinoma	21 (2.7)		18 (1.5)	
Metaplastic carcinoma	8 (1.0)		10 (0.9)	
Tubular carcinoma	2 (0.3)		4 (0.3)	
Other	27 (3.5)		23 (2.0)	
*Molecular subtypes*				
Luminal A	317 (41.0)	< 0.001	620 (52.5)	< 0.001
Luminal B (HER2-)	243 (31.4)		309 (26.2)	
Luminal B (HER2 +)	69 (8.9)		111 (9.4)	
HER2 type	63 (8.1)		45 (3.8)	
Five-negative phenotype	25 (3.2)		28 (2.4)	
Basal phenotype	57 (7.4)		68 (5.8)	
Mitoses/10 HPF, median (IQR p25, p75)	2 (7, 15)		4 (1, 10)	
*Mitoses /10 HPF (%)*				
≤ 2	203 (26.2)	< 0.001	256 (21.8)	< 0.001
> 2, ≤ 5	140 (18.1)		135 (11.5)	
> 5, ≤ 13	202 (26.1)		140 (11.9)	
> 13	229 (29.6)		646 (54.9)	
*Ki-67*				
< 15%	377 (48.7)	< 0.001	680 (57.6)	< 0.001
≤ 15%	397 (51.3)		501 (42.4)	

NOS = Not otherwise specified, HPF = High Power Field

### Characteristics of ER low positive tumours

The distribution of tumour characteristics in patients with ER Low tumours are shown in Table 4. There was a total of 65 (3.3%) ER Low Positive tumours in this study. Of these, 16 were diagnosed before 1995, and 49 was diagnosed in 1995 or later. Among the ER Low Positive tumours diagnosed before 1995, 8/16 (50%) died from BC during follow-up, as opposed to 8/49 (16.3%) of those diagnosed in 1995 or later. Among ER Low tumours, the proportion of tumours < 2 cm, rose from 31% in patients diagnosed before 1995 to 57% in those diagnosed in 1995 or later (*p* < 0.0001).

**Table 4:** Patient and tumour characteristics among patients with ER Low Positive (≥ 1 < 10%) diagnosed before 1995, and in 1995 or later

	Women diagnosed with BC before 1995 (%)	Women diagnosed with BC in 1995 or later (%)	*p*-value
Total cases (*n*)	16	49	
Mean age at diagnosis (SD)	66.9 (12.8)	62.2 (14.2)	
Mean follow-up-time (SD)	10.8 (11.5)	10.2 (4.7)	
Deaths from breast cancer (%)	8 (50.0)	8 (16.3)	< 0.001
Deaths from other causes or by the end of 2015 (%)	7 (43.7)	8 (16.3)	
Alive at end of follow-up	1 (6.3)	33 (67.4)	
*Tumour size*			
≤ 2 cm (%)	5 (31.2)	28 (57.1)	< 0.001
> 2 ≤ 5 cm (%)	1 (6, 3)	14 (28.6)	
Tumour size > 5 cm (%)	0 (0.0)	3 (6.1)	
Uncertain, but > 2 cm (%)	6 (37.5)	1 (2.0)	
Uncertain (%)	4 (25.0)	3 (6.1)	
*Stage*			
1	5 (31.3)	20 (40.8)	0.001
2	2 (12.5)	24 (49.0)	
3	2 (12.5)	1 (2.0)	
4	2 (12.5)	0 (0.0)	
Unknown	5 (31.3)	4 (8.2)	
*Histopathological grade*			
1	0 (0.0)	6 (12.2)	0.041
2	5 (31.2)	26 (53.1)	
3	11 (68.8)	17 (34.7)	
*Molecular subtypes*			
Luminal A	4 (25.0)	25 (51.0)	0.037
Luminal B (HER2-)	4 (25.0)	15 (30.6)	
Luminal B (HER2 +)	8 (50.0)	9 (18.4)	
HER2 type	0 (0.0)	0 (0.0)	
5NP	0 (0.0)	0 (0.0)	
BP	0 (0.0)	0 (0.0)	
Mitoses/10 HPF, median (IQR p25, p75)	9.5 (5, 16.5)	8 (2, 17)	
*Mitoses /10 High power field (HPF) p25* = *4, p50* = *8, p75* = *17 (ER Low)*			
≤ 4/10 HPF	4 (25.0)	8 (16.3)	0.047
> 4 ≤ 8/10 HPF	3 (18.7)	5 (10.2)	
> 8 ≤ 17/10 HPF	5 (31.3)	5 (10.2)	
> 17/10 HPF	4 (25.0)	31 (63.3)	
*Ki-67*			
< 15%	5 (31.2)	26 (53.1)	0.129
≤ 15%	11 (68.8)	23 (46.9)	

For all cases, there was a higher proportion of grade 1 tumours (17.2%), and a lower proportion of tumours with grade 3 (29.6%) among women diagnosed in 1995 or later, compared to women diagnosed before 1995 (Grade 1: 10.9%, Grade 3: 39.1% (*p* < 0.0001)). Among ER Low Positive cases, there was a higher proportion of grade 1 (12.2%) and 2 (53.1%) tumours among women diagnosed in 1995 or later, compared to the women diagnosed before 1995 (grade 1: 0%, grade 2: 31.2%). For grade 3 tumours the proportion of ER low tumours was lower when diagnosed in 1995 or later (*p* = 0.04) (Table 4).

For all cases, the proportion of Luminal A subtype was higher for women diagnosed in 1995 or later (52.5%) compared to those diagnosed before 1995 (41.0%). The proportion of Luminal B (HER2-) and HER2 subtypes was lower for women diagnosed in 1995 or later (*p* < 0.0001) (Table 3), compared to those diagnosed before 1995. Among ER Low Positive tumours, the proportion of Luminal A subtype rose from 25% in ER Low tumours diagnosed before 1995, to 51% when diagnosed in 1995 or later. The proportion of Luminal B (HER2 +) tumours was lower among the women diagnosed in 1995 or later (18.4%), than the women diagnosed before 1995 (50%) (*p* = 0.037) (Table 4).

### ER categories and prognosis

**Fig. 2:**
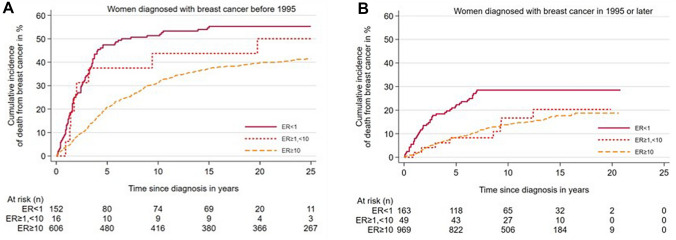
Cumulative incidence of death from breast cancer according to oestrogen receptor (ER) levels. **A** Women diagnosed with BC before 1995. **B** Women diagnosed with BC in 1995 or later. Gray’s test: *p* < 0.0001

Cumulative incidence of death by BC according to ER status is shown in Fig. 2. The risk of death from BC for all categories of ER expression was lower for women diagnosed in 1995 or later compared to women diagnosed before 1995 (Table 5). The cumulative risk of death from BC after 5 years, for women diagnosed before 1995, was 47.4% among cases with ER < 1%, 37.5% for cases with ER Low Positive and 20.8% for cases with ER ≥ 10%. Among women diagnosed with breast cancer in 1995 or later the cumulative risk of death from BC was 22.3% after 5 years for ER < 1%, and 8.3% for both the ER Low Positive and ER ≥ 10% group (Table 5). Thus, among patients diagnosed in 1995 or later, there was no clear difference in risk of death from BC between cases with ER Low Positive and ER > 10%.

**Table 5:** Absolute and relative risk of death from breast cancer according to ER levels, and breast cancer diagnosis before 1995 and in 1995 or later

	ER levels, diagnosis before 1995			ER levels, diagnosis in 1995 or later		
	< 1%	≥ 1 < 10%	≥ 10%	< 1%	≥ 1 < 10%	≥ 10%
Cumulative risk after 5 years (%) (95% CI)	47.4 (39.8–55.6)	37.5 (18.9–65.1)	20.8 (17.8–24.3)	22.3 (16.6–29.5)	8.3 (3.2–20-5)	8.3 (6.8–10.3)
Cumulative risk after 10 years (%) (95% CI)	51.3 (43.7–59.5)	43.8 (23.8–70.5)	31.4 (27.8–35.2)	28.5 (22.1–36.3)	16.7 (8.2–32.2)	13.8 (11.7–16.3)
HR unadjusted (95% CI)	1.0	0.8 (0.4–1.6)	0.6 (0.5–0.7)	1.0	0.5 (0.2–1.0)	0.5 (0.3–0.6)
HR adjusted for age (95% CI)	1.0	0.7 (0.3–1.8)	0.6 (0.4–0.8)	1.0	0.6 (0.3–1.3)	0.4 (0.3–0.6)
HR adjusted for stage (95% CI)	1.0	0.8 (0.3–1.9)	0.6 (0.4–0.7)	1.0	0.6 (0.3–1.2)	0.4 (0.3–0.6)
HR adjusted for grade (95% CI)	1.0	0.7(0.4–1.6)	0.7 (0.5–0.9)	1.0	0.6 (0.3–1.2)	0.6 (0.4–0.8)
HR adjusted for age, stage, and grade (95% CI)	1.0	0.7 (0.3–1.8)	0.7 (0.5–1.0)	1.0	0.9 (0.4–1.9)	0.5 (0.3–0.8)

*ER* Oestrogen receptor, *HR* Hazard ratio, *CI* confidence interval

Cox regression analyses showed that the risk of death was lower among patients with ER ≥ 10%, compared to those with ER < 1%, both among patients diagnosed before 1995, and among patients diagnosed in 1995 or later. The Cox analysis shows a lower relative risk of death from BC among patients with ER ≥ 10% tumours, compared to ER < 1% both before and after 1995. We observed a tendency towards a lower relative risk of death from BC among ER Low Positive, compared to ER < 1%. However, these findings were not statistically significant (Table 5).

## Discussion

In this study of 1955 primary BC tumours, we found that 65 (3.3%) tumours fell under the ER Low Positive category. We found the highest proportion of ER Low Positive among Luminal B (HER2 +) tumours (9.4%). Among cases diagnosed before 1995, 2.1% were ER Low Positive rising to 4.2% among cases diagnosed in 1995 or later. We found an association between ER Low Positive and high histopathological grade, high Ki-67 levels and high mitotic count. However, the results did not show a significant association with prognosis.

Breast cancer survival in Norway has increased since the mid-1990s as seen in the present and other studies [17]. This may be ascribed to earlier detection [18, 19] and improved treatment [6, 20]. The reduced risk of death observed between the two time-periods for all categories of ER expression, probably reflects earlier diagnosis with the introduction of mammography screening and the introduction of adjuvant treatment therapies in the mid-1990s. The change in prognosis observed across time for patients with ER Low Positive tumours may also be attributed to adjuvant therapy other than antihormonal treatment in addition to changing tumour characteristics such as smaller tumour size and lower histopathological grade. However, a drawback of the present study was lack of availability of disease-free survival data.

ER status is an important indicator of prognosis and a predictor of the effect of endocrine treatment. ER signalling is a main driver of proliferation in ER Positive BCs, and inhibition of ER signalling has improved survival among ER Positive BC patients [6, 21]. Studies suggest that selection of patients for endocrine therapy may need to be further personalized [9, 22, 23]. While most ER + BCs have high IHC scores, about 2–3% of cases are ER Low Positive [10, 24, 25]. In the present study, 3.3% of the total number of cases were ER Low Positive. While these tumours are classified within the ER + category, their risk profile appears to be more like that of ER-negative breast cancers [24]. A recent study found no benefit of endocrine therapy in the ER < 10% group compared to the ER > 10% group [25]. The lack of benefit of endocrine therapy in patients with low ER expression has recently been shown in a meta-analysis, including more than 16,000 patients [26]. The meta-analysis indicated that primary BC patients with ER 1–9% gained no significant survival benefit from endocrine therapy, but manifested better overall prognosis than patients with cancers expressing ER < 1% [26]. In the present study, among patients diagnosed in 1995 or later, the ER Low Positive patient group had similar survival to those with ER ≥ 10%. The patients included in this study were diagnosed with BC between 1961 and 2012, and the ER > 1% cut-off level for endocrine treatment was first introduced in Norway in 2011 after recommendations from ASCO/CAP [27]. Therefore, the improved prognosis seen among ER Low Positive patients diagnosed in 1995 or later, can most likely not be attributed to endocrine treatment [28]. Among women diagnosed in 1995 or later, we found a greater proportion of ER Low Positive tumours with smaller size, lower grade, and lower proliferation compared to ER Low Positive tumours diagnosed before 1995. Thus, the improved prognosis may be attributed to factors other than endocrine treatment, such as earlier diagnosis due to the introduction of mammography screening and greater BC awareness among women. Determining endocrine treatment for patients with a diagnosis of ER Low Positive BC should be carefully considered in light of the potential risks and benefits of the treatment [24].

In the present study, the proportion of Luminal A tumours was higher among women diagnosed in the time period during which adjuvant treatment and earlier diagnosis became available, a finding previously observed by our group in an analysis of cohorts 1 and 2 [14]. It has been suggested that BC patients with ER Low Positive are more similar to the ER-negative group, and therefore may not profit from endocrine therapy [9]. Thus, it has been suggested that cut-off levels should be further investigated in order to offer BC patients personalized endocrine treatment [22, 29, 30]. In the present study we found that among cases diagnosed in 1995 or later, ER Low Positive cases showed a prognosis similar to that of ER ≥ 10% cases. However, the impact of hormonal therapy could not be assessed in this study, due to lack of individual information on treatment.

Similar to our findings, a recent study showed that ER Low Positive tumours were more frequently grade 3 and had a higher expression of Ki-67, compared to BCs with intermediate or high expression of ER [31]. Furthermore, they found that the expression of immune-related biomarkers in ER Low Positive was similar to that of ER-negative tumours. We observed four cases of medullary carcinoma and one metaplastic carcinoma among the ER Low Positive cases. When determining treatment for patients with ER Low Positive BC, it may be useful to consider including a panel of immune-related biomarkers.

The FFPE tumour tissue included in this study covered a diagnostic timespan of several decades, and preanalytical conditions may have varied over time. Many of the tumours were diagnosed at a time when ER IHC was not done in the diagnostic setting. However, valuable information can be drawn from archival tissue blocks [32, 33]. It has been shown that antigenicity is, for the most part, preserved in paraffin blocks over decades but may decrease in sections stored over time, resulting in weaker staining [33–35]. We observed no apparent trend towards a negative result among the older specimens but felt it would be unwise to attempt to quantify staining intensity due to the varying preanalytical conditions over which we had no control.

Other strengths of this study include reliable information on BC incidence and follow-up data that were available from high-quality national registries like the Cancer Registry of Norway, the Cause of Death Registry and the Norwegian Patient register [36, 37] thus enabling comparability within the study population across time.

Using TMA sections enables us to stain hundreds of tumour samples at the same time, under the same conditions. The samples comprise a small amount of the original tumour tissue samples, compared to full-face sections. Thus, some important information from the tumour may be lost. However, it has been shown that IHC for ER carried out on sections from TMAs can provide equivalent information regarding clinical endpoint when compared to IHC on full-face tissue Sections [38, 39]. Immunohistochemistry for ER on full-face tissue sections was not carried out in the present study.

## Conclusion

Overall, ER Low Positive BCs exhibited many characteristics similar to ER-negative tumours and were frequently Luminal B (HER2 +). Among women diagnosed in 1995 or later, the proportion of ER Low Positive BCs was higher than among women diagnosed before 1995 and ER Low Positive tumours diagnosed in 1995 or later were of smaller size, lower grade, lower proliferative status, and were more frequently Luminal A Women with ER Low Positive tumours had similar prognosis to patients with ER ≥ 10% when diagnosed in 1995 or later.

## Data Availability

The datasets generated and/or analysed during this study are not publicly available due to issues of sensitivity and limitations determined in the conditions for approval by the Regional Committee for Medical and Health Research Ethics. However, the data may be made available from the corresponding author on reasonable request.
